# Early wound closure with negative-pressure irrigation-drainage accelerates healing and improves early appearance: a comparative study

**DOI:** 10.3389/fsurg.2026.1851638

**Published:** 2026-07-28

**Authors:** Yan Nie, Quanli Geng, Shuang Jin, Zhi Liu, Xu Ma, Jinsheng Ye

**Affiliations:** 1 Department of General Surgery, Yanqing Hospital of Beijing Chinese Medicine Hospital, Beijing, China; 2 Department of General Surgery, Beijing Hospital of Traditional Chinese Medicine, Capital Medical University, Beijing, China

**Keywords:** comparative study, early wound appearance, early wound closure, negative-pressure irrigation-drainage, wound healing

## Abstract

**Objective:**

Delayed wound healing remains a common clinical challenge and is associated with increased infection risk, prolonged hospitalization, and poor scar outcomes. Whether a multimodal strategy comprising early wound closure supported by continuous negative-pressure irrigation and drainage can improve healing dynamics compared with conventional open management remains insufficiently explored.

**Methods:**

This comparative clinical study included 62 patients with wounds who were admitted to Yanqing Hospital of Beijing Chinese Medicine Hospital between January and December 2023. Thirty-two patients received conventional open wound care (control group), and 30 patients underwent early primary closure with post-operative continuous negative-pressure irrigation and drainage (intervention group).Baseline characteristics, clinical outcomes, and wound healing trajectories were analyzed. Time to wound healing was evaluated using Kaplan–Meier analysis and Cox proportional hazards models. Multivariable logistic regression analyses were performed to assess wound healing within 14 and 28 days after adjustment for potential confounders.

**Results:**

Patients in the intervention group exhibited a significantly shorter median wound healing time compared with controls (17.5 vs. 24.5 days, *P* = 0.003). The cumulative probability of wound healing was significantly higher in the intervention group(log-rank *P* = 0.003), and this association remained significant after multivariable adjustment (adjusted HR = 3.17, 95% CI: 1.66–6.05). Although no significant difference in healing within 14 days was observed after adjustment, the intervention was associated with a higher likelihood of complete wound healing within 28 days (adjusted OR = 11.63, 95% CI: 1.06–127.72). In addition, the intervention was associated with faster wound area reduction and better early wound cosmetic scores.

**Conclusions:**

A multimodal strategy consisting of early primary closure supported by continuous negative-pressure irrigation and drainage was associated with accelerated wound healing and improved early wound appearance. E-value analysis suggested that unmeasured confounding is unlikely to fully explain the observed association, though residual confounding from selection bias cannot be excluded.No increase in adverse events was observed. Due to the retrospective design and relatively small sample size, these findings are hypothesis-generating. Prospective randomized trials are required to establish definitive clinical recommendations.

## 1 Introduction

Delayed wound healing remains a common and clinically significant challenge in surgical and traumatic wounds, particularly in the presence of contamination or local infection. Prolonged healing not only increases the risk of secondary infection and adverse scarring but also imposes substantial physical and psychological burdens on patients, as well as increased healthcare costs (1, 2). Conventional wound management strategies often emphasize delayed closure or secondary intention healing for contaminated wounds, aiming to reduce the risk of infection. However, prolonged open wound management may paradoxically exacerbate inflammation, delay granulation, and worsen cosmetic outcomes (3, 4). In recent years, increasing attention has been directed toward optimized wound irrigation techniques and timely wound closure as potential strategies to promote faster healing while maintaining infection control (5, 6). Adequate irrigation plays a critical role in reducing bacterial load, removing devitalized tissue, and modulating the wound microenvironment (7). When combined with early wound closure under appropriate clinical conditions, irrigation may facilitate rapid epithelialization, reduce inflammatory burden, and improve both functional and aesthetic outcomes (8). Nevertheless, concerns persist regarding the safety of early closure, particularly with respect to infection recurrence and wound complications, and robust comparative clinical data remain limited.

This study aimed to evaluate the effectiveness and safety of a multimodal strategy consisting of early primary closure supported by continuous negative-pressure irrigation and drainage, compared with conventional open wound management in patients with contaminated or infected wounds.Specifically, we assessed time to wound healing, healing rates at predefined time points, inflammatory markers, wound size dynamics, pain intensity, early wound appearance, and adverse events.We further employed multivariable regression analyses to examine whether this multimodal strategy independently influenced wound healing outcomes after adjustment for potential confounders.

## 2 Methods

### 2.1 Study design and participants

This retrospective comparative cohort study was conducted at Yanqing Hospital of Beijing Chinese Medicine Hospital and included consecutive adult patients with infected, open, or traumatic wounds treated between January and December 2023.

Patients were eligible for inclusion if they met the following criteria: (1) age ≥18 years; (2) diagnosis of infected or contaminated cutaneous wounds; (3) wound area between 3 and 20 cm² at baseline; (4) injury occurring within 72 h prior to admission; (5) availability of complete clinical data and follow-up until wound healing.

Exclusion criteria were as follows: (1) wounds with exposure of major blood vessels or vital organs; (2) wounds communicating with internal organs via fistula formation; (3) presence of severe cardiac, hepatic, or renal dysfunction, hematologic diseases, malignancy, or other systemic conditions known to impair wound healing; (4) known allergy to suture materials; (5) incomplete clinical data or insufficient follow-up for outcome assessment.

“Contaminated” wounds were defined as those containing foreign material or devitalized tissue without overt purulence. “Infected” wounds were defined as those with purulent discharge, surrounding erythema, local warmth, or positive wound culture.

A total of 62 patients met the eligibility criteria and were included in the final analysis.

### 2.2 Treatment allocation and interventions

Patients were allocated to one of two groups according to the wound management strategy applied. Treatment allocation was determined by the attending surgeon's clinical judgment. Patients were assigned to the intervention group if the wound was deemed suitable for primary closure after debridement (e.g., adequate tissue perfusion, absence of gross purulence or deep necrotic tissue, ability to achieve tension-free closure). The control group comprised patients who received standard open wound management, either because the wound was not considered suitable for immediate closure or as per the surgeon's preference.

#### 2.2.1 Control group

Patients in the control group received standard wound care according to institutional protocols. Briefly, wounds were irrigated with sterile normal saline to remove surface contaminants and foreign material, followed by disinfection of the surrounding skin with povidone–iodine. For wounds with purulent discharge, hydrogen peroxide was used for initial irrigation, followed by thorough saline irrigation. Appropriate dressings (e.g., petrolatum gauze or povidone–iodine gauze) were applied based on wound characteristics. Dressing changes were performed at intervals determined by wound exudate and infection status until complete healing was achieved. Wounds healed by secondary intention.

#### 2.2.2 Intervention group (early closure with continuous negative-pressure irrigation and drainage)

**Figure 1:**
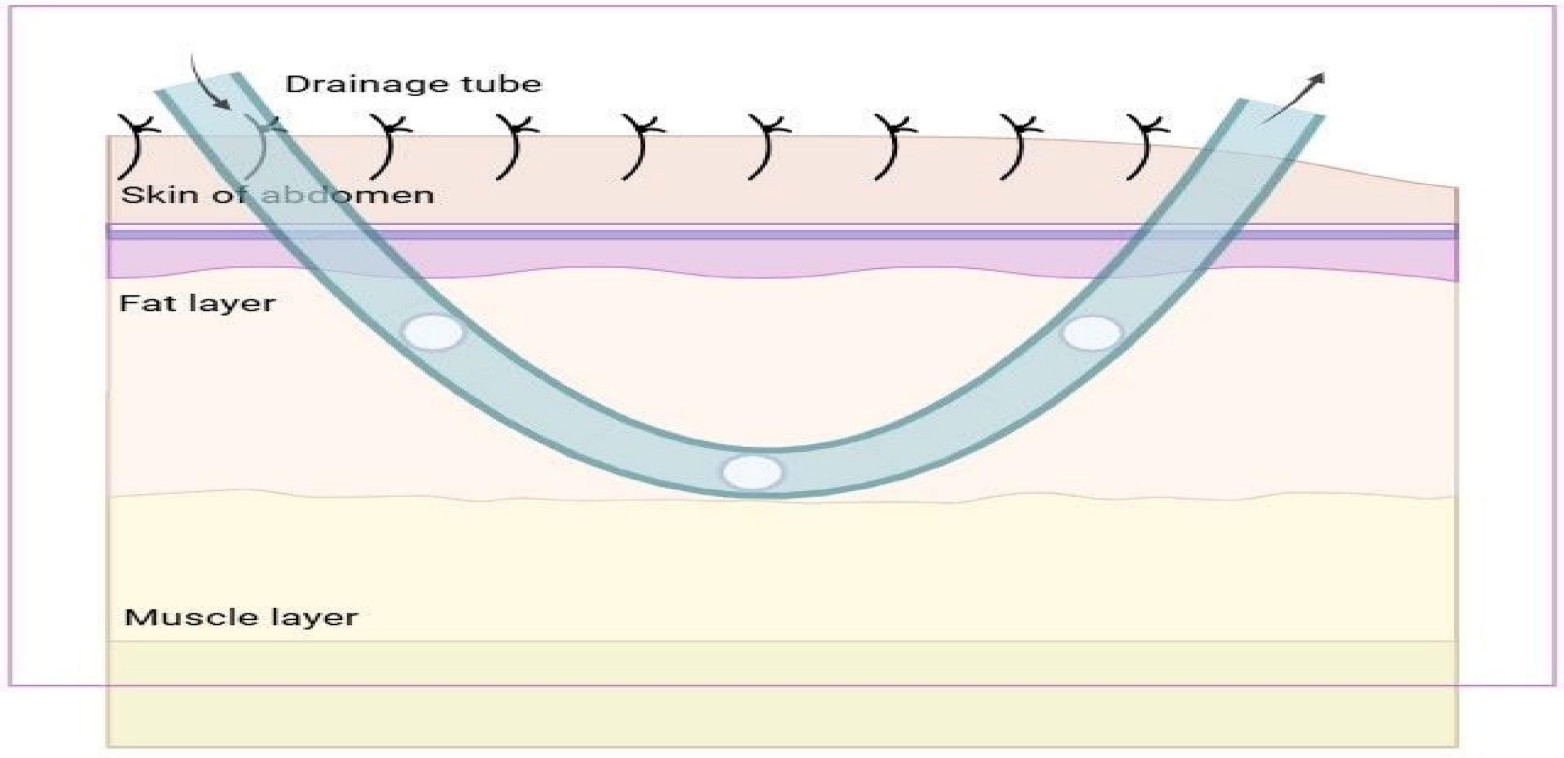
Schematic illustration of the intervention procedure (early closure with continuous negative-pressure irrigation and drainage).

**Figure 2:**
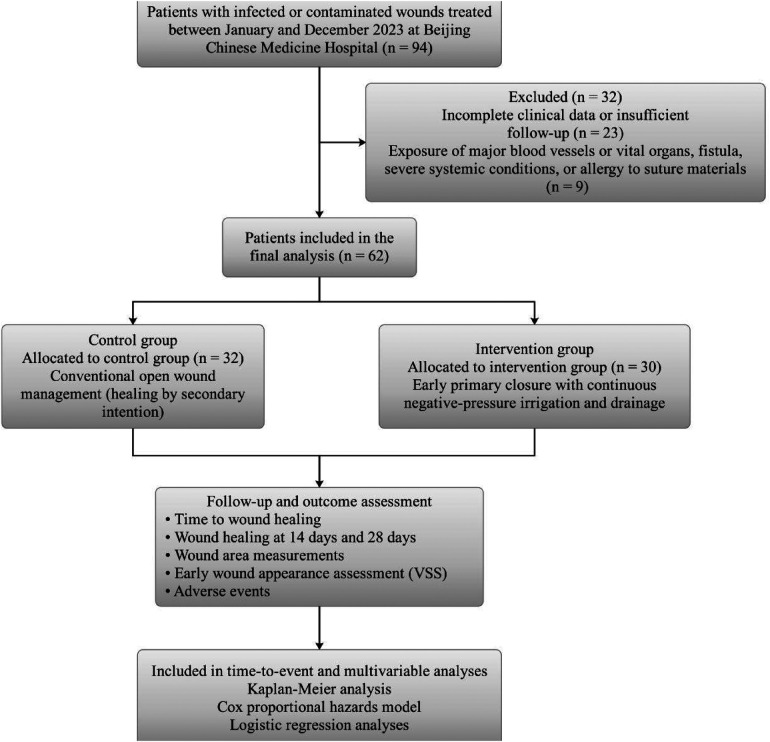
Flow diagram of patient selection and study design.

Patients in the intervention group underwent early surgical debridement, wound irrigation, primary closure, and continuous drainage when clinically appropriate. After positioning to fully expose the wound, the surrounding skin was disinfected using 0.5% povidone–iodine and sterile draping was applied. The wound was thoroughly irrigated with sterile normal saline to remove debris, foreign material, and necrotic tissue. Deep wounds were additionally irrigated with hydrogen peroxide to eliminate anaerobic pathogens. Hemostasis was meticulously achieved, and wound exudate or tissue samples were collected for bacterial culture and antimicrobial susceptibility testing when clinical signs of infection were present (e.g., purulent discharge, necrotic tissue, or surrounding cellulitis).A drainage tube (16–20 Fr) with multiple lateral side holes was placed in the deep portion of the wound to ensure adequate drainage. The wound was closed with interrupted simple sutures (3–0 or 4–0 nylon or absorbable suture) placed approximately 0.5–1 cm apart, avoiding excessive tension. After closure, the wound was disinfected again and covered with sterile dressings. One end of the drainage tube was connected to an infusion set and the other end to a negative-pressure suction device. The infusion system delivered sterile saline combined with gentamicin (80 mg/L) as empirical antibiotic therapy. In patients with positive wound cultures, the antibiotic was adjusted according to susceptibility results. The negative-pressure suction device was set at −80 to −120 mmHg continuously. The infusion rate was approximately 40–50 drops per minute. Daily input and output volumes were recorded, and dressing changes were performed accordingly. The irrigation rate was adjusted based on the appearance and volume of the drainage fluid. Continuous irrigation was discontinued and the drainage tube was removed when drainage volume markedly decreased, drainage fluid became clear, local skin temperature normalized, signs of infection resolved, and no significant tenderness was present upon palpation. A schematic illustration of the key procedural steps involved in the intervention—including wound disinfection, debridement, drainage tube placement, wound closure, and postoperative irrigation—is shown in Figure 1. The overall study design and patient selection process are summarized in Figure 2.

### 2.3 Outcomes

The primary outcome was time to wound healing, defined as the number of days from initial treatment to complete epithelialization. Complete epithelialization was assessed visually by a wound care nurse who was not involved in treatment allocation and was unaware of group assignment. The criterion for complete epithelialization was that the entire wound surface was covered by continuous new epithelium without erosion or exudate. The first day on which these criteria were met was recorded. Digital photographs were taken at each assessment for retrospective confirmation. Follow-up assessments were conducted every 2–3 days during the first two weeks and then weekly until healing. Secondary outcomes included wound healing within 14 and 28 days, wound area measurements at baseline and postoperative days 3, 7, and 10, inflammatory markers (WBC count and CRP), pain intensity assessed using the visual analogue scale (VAS), wound discharge and erythema grades, early wound cosmetic quality assessed using the Vancouver Scar Scale at the time of wound closure (approximately day 28), patient satisfaction, and adverse events.

### 2.4 Statistical analysis

Continuous variables were summarized as mean ± standard deviation (SD) or median (interquartile range, IQR), as appropriate. Between-group comparisons were performed using the independent-samples t test for normally distributed variables and the Mann–Whitney *U*-test for non-normally distributed variables. Categorical variables were expressed as counts (percentages) and compared using the chi-square test or Fisher's exact test, as appropriate.Standardized mean differences (SMD) were calculated for baseline variables; an absolute SMD >0.1 was considered indicative of meaningful imbalance. Time to wound healing was analyzed using Kaplan–Meier survival curves and compared between groups using the log-rank test. Cox proportional hazards regression models were constructed to estimate hazard ratios (HRs) with 95% confidence intervals (CIs). The proportional hazards assumption was tested using Schoenfeld residuals. Logistic regression analyses were conducted to evaluate wound healing outcomes at 14 and 28 days. Variables with clinical relevance or those showing potential associations in univariable analyses were included in multivariable models. All statistical tests were two-sided, and a *P* value < 0.05 was considered statistically significant. Statistical analyses were performed using R software (version 4.1.0; R Foundation for Statistical Computing, Vienna, Austria). Graphical visualizations were generated using GraphPad Prism software (version 8.0; GraphPad Software, San Diego, CA, USA).

## 3 Results

### 3.1 Baseline characteristics

A total of 62 patients were included in the analysis, with 32 patients in the control group and 30 patients in the intervention group. Baseline characteristics are summarized in Table 1. Patients in the intervention group were significantly younger than those in the control group (39.87 ± 7.04 vs. 47.94 ± 13.18 years, *P* = 0.004; SMD = 0.72) and had smaller baseline wound areas (5.93 ± 2.43 vs. 7.53 ± 2.12 cm^2^, *P* = 0.008; SMD = 0.68). No significant differences were observed between the two groups with respect to baseline WBC count, baseline CRP level, sex distribution, or the prevalence of diabetes (all *P* > 0.05). The distribution of wound etiologies, anatomic locations, and time from injury to treatment was broadly comparable between groups (Supplementary Table S1).

**Table 1:** Baseline characteristics of patients in the control and the intervention group.

Variables	Total (*n* = 62)	Control (*n* = 32)	Intervention (*n* = 30)	*P*	SMD
Age, Mea*n* ± SD	44.03 ± 11.33	47.94 ± 13.18	39.87 ± 7.04	0.004	0.72
WBC baseline, Mean ± SD	8.61 ± 2.96	8.63 ± 3.05	8.59 ± 2.91	0.958	0.01
Wound area baseline, Mean ± SD	6.76 ± 2.40	7.53 ± 2.12	5.93 ± 2.43	0.008	0.68
CRP baseline, M (Q₁, Q₃)	15.85 (8.83, 24.32)	18.15 (9.47, 31.70)	12.60 (8.27, 20.41)	0.076	0.32
Sex, n(%)				0.632	0.12
Female	27 (43.55)	13 (40.62)	14 (46.67)		
Male	35 (56.45)	19 (59.38)	16 (53.33)		
Diabetes, n(%)				1.000	0.00
No	31 (50.00)	16 (50.00)	15 (50.00)		
Yes	31 (50.00)	16 (50.00)	15 (50.00)		

Continuous variables are presented as mean ± standard deviation (SD) or median (interquartile range, IQR), as appropriate.

Categorical variables are presented as number (percentage).

Between-group comparisons were performed using the independent-samples *t*-test for normally distributed continuous variables, the Mann–Whitney *U*-test for non-normally distributed variables, and the chi-square test or Fisher's exact test for categorical variables. SMD, standardized mean difference. An absolute SMD >0.1 was considered indicative of meaningful between-group imbalance. A two-sided *P* value < 0.05 was considered statistically significant.

### 3.2 Clinical outcomes

Clinical outcomes are presented in Table 2. The intervention group demonstrated significantly improved wound healing outcomes compared with the control group. The median wound healing time was significantly shorter in the intervention group than in the control group (17.5 [14.0–22.0] days vs. 24.5 [21.0–28.0] days, *P* = 0.003). In addition, a significantly higher proportion of patients in the intervention group achieved wound healing within 14 days (36.67% vs. 15.62%, *P* = 0.04) and within 28 days (96.67% vs. 75.00%, *P* = 0.02). With respect to inflammatory markers, no statistically significant differences were observed between the two groups in WBC count at postoperative day 7 (6.79 ± 0.59 vs. 7.58 ± 2.34 × 10^9^/L, *P* = 0.100) or CRP level at day 7 (4.80 [4.27–26.70] vs. 8.50 [4.70–12.10] mg/L, *P* = 0.853). Wound area measurements were significantly smaller in the intervention group at postoperative day 3 and day 7 (both *P* < 0.001), and the mean wound area at day 10 was also significantly reduced compared with the control group (2.54 ± 1.36 vs. 5.26 ± 1.06 cm^2^, *P* < 0.001). Pain intensity assessed by the visual analogue scale (VAS) did not differ significantly between the two groups on postoperative day 1 or day 7. However, patients in the intervention group reported significantly lower pain scores on postoperative day 3 (*P* = 0.032). Furthermore, wound discharge grade, erythema range grade, and early wound scores assessed using the Vancouver Scar Scale were all significantly lower in the intervention group (all *P* ≤ 0.002). No significant differences were observed between the two groups in patient satisfaction rates or the incidence of adverse events.

**Table 2:** Clinical outcomes in the control and intervention groups.

Outcome	Control (*n* = 32)	Intervention (*n* = 30)	*P* value
Wound healing time (days)	24.5 (21.0–28.0)	17.5 (14.0–22.0)	0.003
Healed_14d, (*n*, %)	5 (15.62)	11 (36.67)	0.04
Healed_28d, (*n*, %)	24 (75.0)	29 (96.67)	0.02
WBC day 7 (×10^9^/L), mean ± SD	7.58 ± 2.34	6.79 ± 0.59	0.100
CRP day 7 (mg/L) median (IQR)	8.50 (4.70–12.10)	4.80 (4.27–26.70)	0.853
Wound area day 3 (cm^2^)	8.00 (6.00–8.00)	5.00 (4.00–6.00)	<0.001
Wound area day 7 (cm^2^)	7.00 (6.00–7.00)	4.00 (3.00–5.00)	<0.001
Wound area day 10 (cm²)	5.26 ± 1.06	2.54 ± 1.36	<0.001
VAS day 1	5.00 (5.00–6.00)	5.00 (5.00–6.00)	0.883
VAS day 3	3.00 (3.00–4.00)	3.00 (2.00–3.00)	0.032
VAS day 7	2.00 (1.00–2.00)	2.00 (1.00–2.00)	0.695
Wound discharge grade (0–2)	1.00 (0.00–2.00)	0.00 (0.00–1.00)	0.002
Erythema range grade (0–3)	1.00 (0.00–2.00)	0.00 (0.00–1.00)	<0.001
VSS early wound score	5.00 (4.00–5.00)	2.00 (2.00–2.00)	<0.001
Patient satisfaction, (*n*, %)	17 (56.67)	22 (68.75)	0.146
Adverse events (*n*, %)	8 (25)	7 (23.33)	0.878

Continuous variables are presented as mean ± standard deviation (SD) or median (interquartile range, IQR), as appropriate.

Categorical variables are presented as number (percentage).

Between-group comparisons were performed using the independent-samples *t*-test or Mann–Whitney *U*-test for continuous variables, and the chi-square test or Fisher's exact test for categorical variables.

Time to wound healing was compared using the log-rank test.

VSS, Vancouver Scar Scale (assessed at wound closure, approximately day 28).

A two-sided *P* value < 0.05 was considered statistically significant.

### 3.3 Time-to-wound healing analysis

**Figure 3:**
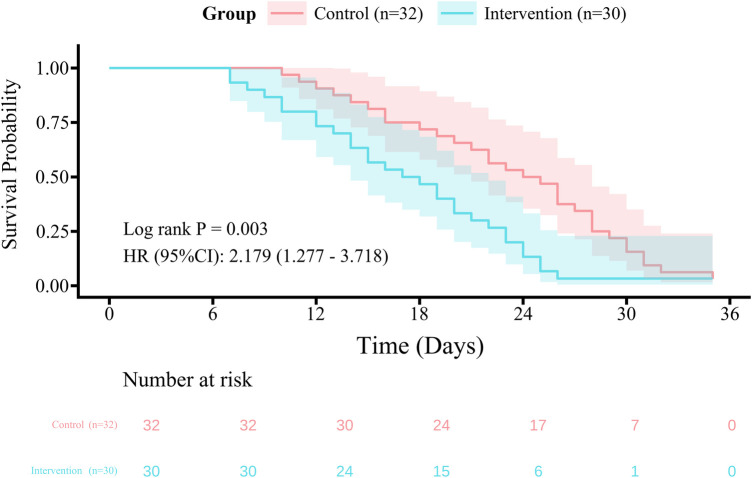
Kaplan–meier analysis of time to wound healing between the two treatment groups.

Kaplan–Meier analysis revealed a significant difference in time to wound healing between the two treatment groups (Figure 3). The cumulative probability of remaining unhealed declined more rapidly in the intervention group throughout the follow-up period. The log-rank test demonstrated a statistically significant difference in healing time distributions between the two groups (*P* = 0.003). In the unadjusted Cox proportional hazards analysis, the intervention was associated with a significantly higher wound healing rate compared with the control group (HR = 2.18, 95% CI: 1.28–3.72).

### 3.4 Changes in wound area over time

**Figure 4:**
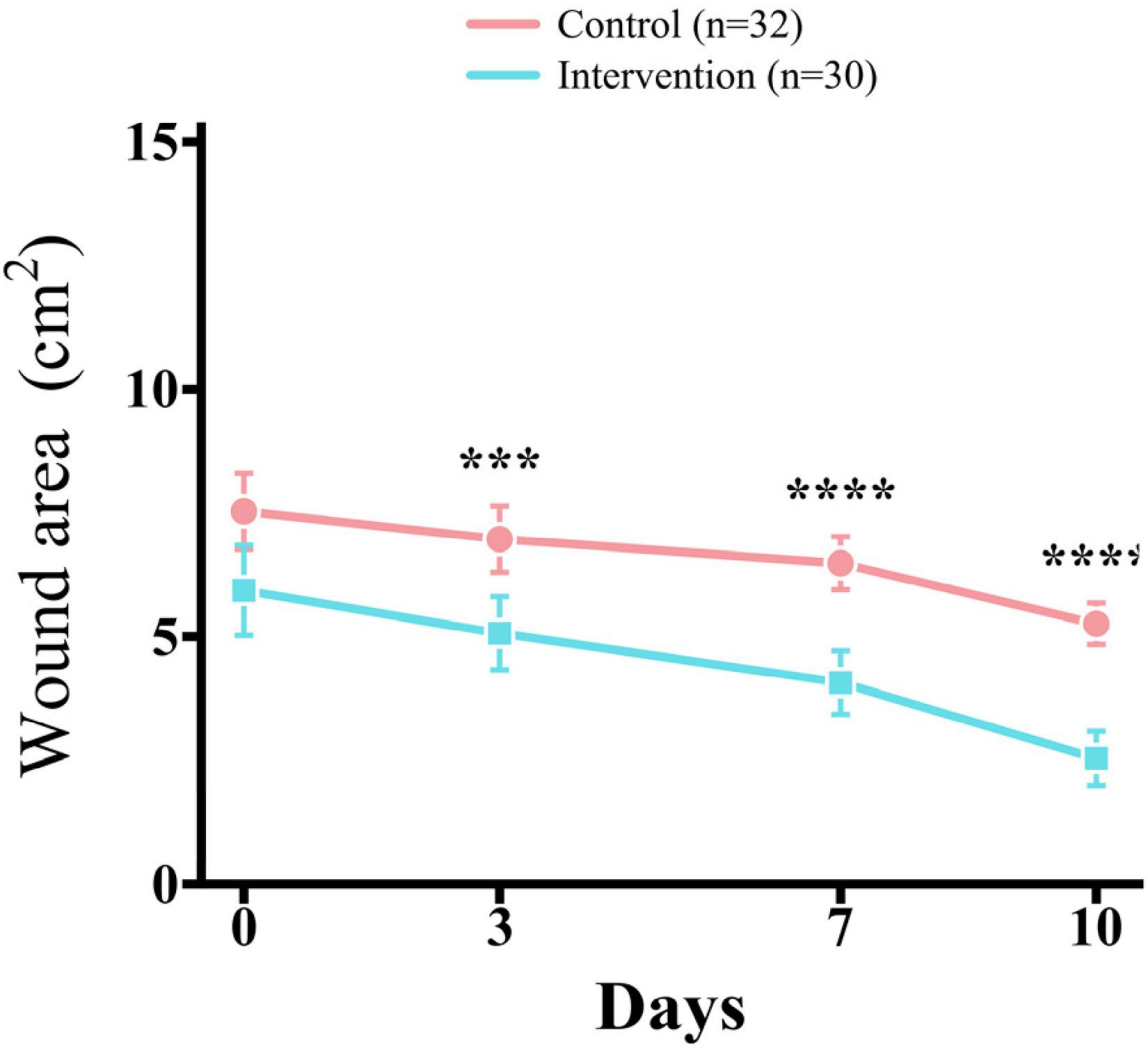
Changes in wound area over time between the two groups.

In addition to the cross-sectional comparisons presented in Table 2, longitudinal changes in wound area were further examined (Figure 4). The intervention group exhibited a more pronounced and sustained reduction in wound size over time. Specifically, wound area was significantly smaller in the intervention group at postoperative day 3, day 7, and day 10 compared with the control group (all *P* < 0.001). Despite a smaller baseline wound area in the intervention group, the reduction in wound size was more pronounced at all time points (Figure 4; data presented with 95% confidence intervals).

### 3.5 Early wound appearance

**Figure 5:**
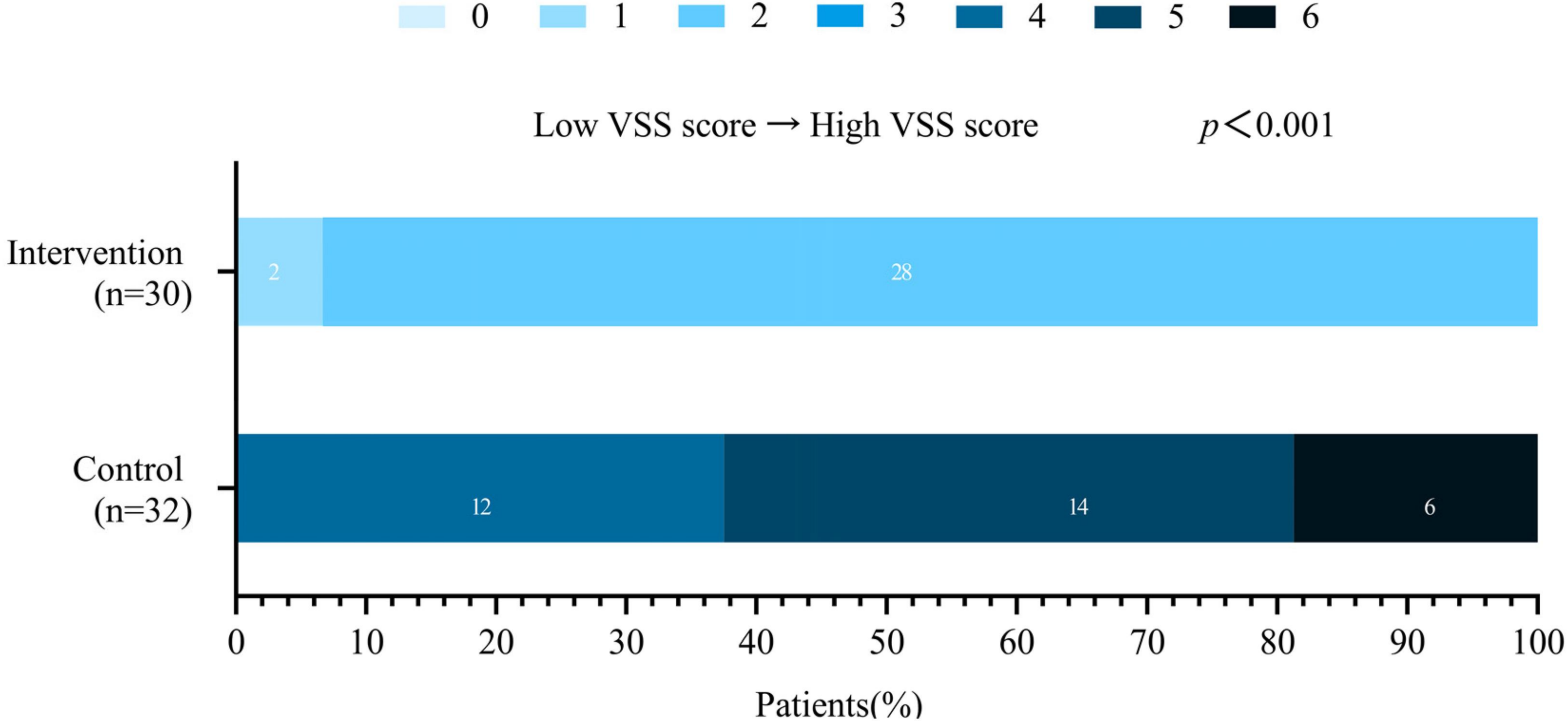
Distribution of early wound cosmetic scores (VSS) between groups.

Early wound appearance, assessed using the Vancouver Scar Scale at the time of wound closure (approximately day 28), is shown in Figure 5. The distribution of scores was shifted toward lower values in the intervention group (*P* < 0.001), indicating better early wound cosmetic quality. These findings reflect early wound appearance rather than mature scar outcomes.

### 3.6 Microbiological findings

Intraoperative wound cultures were obtained in the intervention group when clinical signs of infection were present. Common isolates included Staphylococcus aureus and Pseudomonas aeruginosa; when cultures were positive, the antibiotic in the irrigation solution was adjusted according to susceptibility. Wound cultures were not routinely performed in the control group. Due to the retrospective nature of data collection, a complete microbiological profile cannot be provided for either group.

### 3.7 Multivariable regression analyses of wound healing outcomes

Building on the unadjusted comparisons presented in Table 2, univariable regression analyses were first conducted to identify potential factors associated with wound healing outcomes (Supplementary Tables S2, S3). Variables showing clinical relevance or a potential association with wound healing in univariable analyses were subsequently included in multivariable models.

To further evaluate the independent association between treatment strategy and wound healing outcomes, multivariable regression analyses were performed after adjustment for potential confounders (Tables 3, 4). In the multivariable Cox proportional hazards model for time to wound healing (Table 3), the intervention remained independently associated with a significantly higher healing rate (adjusted HR = 3.17, 95% CI: 1.66–6.05, *P* < 0.001). Diabetes was independently associated with delayed wound healing (adjusted HR = 0.24, 95% CI: 0.12–0.50, *P* < 0.001), whereas age, sex, and baseline wound area were not significantly associated with healing time after adjustment. Multivariable logistic regression analyses were conducted to assess wound healing at fixed time points (Table 4). After adjustment, no statistically significant difference in the likelihood of wound healing within 14 days was observed between the two groups (adjusted OR = 4.23, 95% CI: 0.74–24.25, *P* = 0.105). In contrast, patients in the intervention group exhibited a significantly higher probability of achieving complete wound healing within 28 days compared with those in the control group (adjusted OR = 11.63, 95% CI: 1.06–127.72, *P* = 0.045). Age, diabetes status, and baseline wound area were not independently associated with wound healing at 28 days in the adjusted model.

**Table 3:** Cox proportional hazards analysis for time to wound healing.

Variables	Adjusted HR (95%CI)	*P*
Group		
Control	1.00 (Reference)	
Intervention	3.17 (1.66–6.05)	<.001
Age	0.98 (0.95–1.01)	0.225
Sex		
Female	1.00 (Reference)	
Male	0.67 (0.39–1.15)	0.142
Diabetes		
No	1.00 (Reference)	
Yes	0.24 (0.12–0.50)	<.001
Wound area baseline	1.02 (0.89–1.16)	0.768

Hazard ratios (HRs) were estimated using a multivariable Cox proportional hazards model adjusted for age, sex, diabetes status, and baseline wound area. HR >1 indicates a higher rate of wound healing.

**Table 4:** Logistic regression analysis for wound healing outcomes.

Outcome	Variables	Adjusted OR (95%CI)	*P*
Healed within 14 days	Group		
	Control	1.00 (Reference)	
	Intervention	4.23 (0.74–24.25)	0.105
	Age	1.02 (0.90–1.15)	0.804
	Diabetes		
	No	1.00 (Reference)	
	Yes	–	–
	Wound area baseline	0.82 (0.58–1.17)	0.271
Healed within 28 days	Group		
	Control	1.00 (Reference)	
	Intervention	11.63 (1.06–127.72)	0.045
	Age	0.97 (0.89–1.05)	0.420
	Diabetes		
	No	1.00 (Reference)	
	Yes	0.24 (0.03–2.11)	0.196
	Wound area baseline	1.29 (0.85–1.97)	0.227

Odds ratios (ORs) were estimated using multivariable logistic regression models adjusted for age, diabetes status, and baseline wound area. OR >1 indicates a higher likelihood of wound healing at the specified time point. The diabetes variable was omitted from the 14-day multivariable model due to sparse events.

## 4 Discussion

In this retrospective comparative cohort study, a multimodal strategy consisting of early primary closure supported by continuous negative-pressure irrigation and drainage was associated with faster wound healing, greater wound area reduction, and better early wound appearance compared with conventional wound management, without an increased risk of adverse events.

A key concern in observational studies is the potential for selection bias. In the present study, patients in the intervention group were significantly younger (SMD = 0.72) and had smaller baseline wound areas (SMD = 0.68), reflecting real-world clinical decision-making in which surgeons preferentially offer early closure to patients with more favorable wound characteristics. Both SMD values substantially exceed the conventional threshold of 0.1 for meaningful imbalance. After multivariable adjustment for these measured variables, the association between the intervention and faster healing persisted. However, residual confounding from unmeasured variables—such as severity of wound contamination, tissue perfusion quality, nutritional status, or surgeon experience—cannot be excluded. This baseline imbalance represents a major limitation and precludes causal inference.

To evaluate the potential impact of unmeasured confounding on our primary finding, we performed an E-value analysis for the association between the intervention and time to wound healing (HR = 3.17, 95% CI: 1.66–6.05). The calculated E-value was 5.8 for the point estimate and 2.7 for the lower bound of the 95% confidence interval. These values mean that an unmeasured confounder would need to be associated with both treatment assignment and wound healing by a risk ratio of at least 2.7 (for the lower CI limit) to shift the confidence interval to include the null, after accounting for measured covariates. However, E-value analysis has important limitations in the context of this study. It does not compensate for the measured baseline imbalances (SMD values up to 0.72) that arise from non-randomized treatment allocation. Furthermore, E-value calculations assume that unmeasured confounders are independent of measured confounders, which may not hold in clinical practice. Therefore, E-value results should be viewed as a supplementary sensitivity assessment rather than as evidence supporting the robustness of the findings or as a justification for causal claims (14).

Notably, although the intervention group presented with more favorable baseline characteristics (younger age and smaller wound area), the significant association persisted after multivariable adjustment for these factors. Diabetes emerged as a strong independent predictor of delayed wound healing, consistent with prior literature emphasizing impaired microcirculation and altered inflammatory responses in diabetic patients (9, 10). This pattern suggests that the intervention may exert cumulative benefits over time rather than producing immediate effects, aligning with biological processes of granulation, epithelialization, and tissue remodeling (11).

Regarding wound appearance assessment, the Vancouver Scar Scale was applied at the time of wound closure (approximately day 28) to assess early wound cosmetic quality. We emphasize that the VSS was originally developed to evaluate mature scars, and its application at day 28 captured early wound appearance rather than final scar outcomes. True scar remodeling continues for months after epithelialization, and longer follow-up (≥6 months) would be required to evaluate mature scar formation. Therefore, the results reported in this study should be interpreted as reflecting improved early wound appearance, not improved scar outcomes.This may be attributed to reduced inflammatory burden, earlier wound edge approximation, and more favorable collagen remodeling (12, 13).

The safety profile of the intervention was comparable to conventional management, with no significant difference in adverse event rates (23.33% vs. 25.00%, *P* = 0.878). This is an important finding, as concerns about infection and wound complications have historically limited the adoption of early closure for contaminated or infected wounds. Our results suggest that the intervention included local gentamicin irrigation and culture-guided antibiotic adjustment. However, data on systemic antibiotic use were not systematically recorded and could not be retrieved. Differences in systemic antibiotic therapy between groups cannot be ruled out and may have contributed to the observed outcomes.

Several limitations of this study warrant consideration. First, the retrospective, non-randomized design introduces potential selection bias, as discussed above. Although multivariable adjustment and E-value analysis were applied, residual confounding may still exist. Second, the sample size is relatively modest (*n* = 62), which limits statistical power and resulted in wide confidence intervals for some estimates. Third, the 14-day healing model could not adjust for diabetes due to sparse events. Fourth, the single-center design may limit generalizability. Fifth, wound appearance was assessed at an early time point without long-term follow-up, and the assessor, while not the treating surgeon, was not formally blinded. Sixth, treatment allocation was determined by surgeons based on clinical judgment, which is the primary source of selection bias. Seventh, the intervention group received a combination of early surgical debridement, primary closure, continuous negative-pressure drainage, and local antibiotic irrigation. The independent contribution of each component cannot be isolated, and the observed benefits reflect the overall effect of this combined strategy rather than the specific effect of any single element. Eighth, data on systemic antibiotic use were not systematically documented and could not be retrieved. Ninth, wound cultures were not routinely performed in the control group, and in the intervention group were obtained only when clinical signs of infection were present; a complete and comparable microbiological profile is therefore unavailable.

Despite these limitations, this study has several strengths. Importantly, this study provides real-world benchmark data—including effect sizes, healing trajectories, and safety signals—that can inform the design of future prospective trials. The comprehensive assessment of clinical outcomes, including time-to-event analysis, longitudinal wound area measurements, pain scores, and early wound appearance, offers a multidimensional evaluation of treatment efficacy. The multimodal strategy evaluated reflects how these techniques are actually combined in surgical practice.

## 5 Conclusion

In this retrospective comparative cohort study, a multimodal strategy consisting of early primary closure supported by continuous negative-pressure irrigation and drainage was associated with accelerated wound healing, enhanced wound area reduction, and improved early wound appearance compared with conventional open wound management in selected patients with contaminated or infected wounds. No increase in adverse events was observed, suggesting an acceptable safety profile.

Due to the retrospective design, non-randomized treatment allocation, small sample size (*n* = 62), and substantial baseline imbalances between groups (age SMD = 0.72, wound area SMD = 0.68), these findings should be regarded as hypothesis-generating. The observed associations cannot be interpreted as causal effects. Furthermore, the independent contributions of early closure, negative-pressure drainage, and local antibiotic irrigation cannot be separated in the current study design.

Prospective, multicenter, randomized controlled trials with factorial designs, adequate sample sizes, long-term follow-up for mature scar assessment, and standardized antibiotic protocols are required to validate these findings and to determine the specific contributions of each intervention component.

## Data Availability

The raw data supporting the conclusions of this article will be made available by the authors, without undue reservation.
