# Supplementary material for: Early wound closure with negative-pressure irrigation-drainage accelerates healing and improves early appearance: a comparative study
Source: Front Surg. 2026 Jul 28;13:1851638. doi: 10.3389/fsurg.2026.1851638 (PMC13457209; doi:10.3389/fsurg.2026.1851638)
Supplement: Supplementary file 1 [file Table1.docx]

**Supplementary Table S1. Wound characteristics by group**

| **Characteristic** | **Control (n=32)** | **Interventionon (n=30)** | **P value** |
| --- | --- | --- | --- |
| Wound etiology, n (%) |  |  | 0.58 |
| Postoperative infection | 12 (37.5) | 8 (26.7) |  |
| Pilonidal sinus | 4 (12.5) | 8 (26.7) |  |
| Pressure ulcer | 6 (18.8) | 4 (13.3) |  |
| Abscess rupture | 6 (18.8) | 6 (20.0) |  |
| Ostomy hernia repair | 3 (9.4) | 3 (10.0) |  |
| Other (trauma) | 1 (3.1) | 1 (3.3) |  |
| Anatomic location, n (%) |  |  | 0.48 |
| Sacrococcygeal | 8 (25.0) | 12 (40.0) |  |
| Abdominal | 12 (37.5) | 8 (26.7) |  |
| Lower extremity | 8 (25.0) | 6 (20.0) |  |
| Upper extremity | 3 (9.4) | 3 (10.0) |  |
| Other | 1 (3.1) | 1 (3.3) |  |
| Time from injury to treatment (hours), median (IQR) | 24 (12-48) | 18 (10-36) | 0.18 |

Note: P values were calculated using Fisher's exact test for categorical variables and Mann-Whitney U test for continuous variables.
