# Supplementary material for: Early wound closure with negative-pressure irrigation-drainage accelerates healing and improves early appearance: a comparative study
Source: Front Surg. 2026 Jul 28;13:1851638. doi: 10.3389/fsurg.2026.1851638 (PMC13457209; doi:10.3389/fsurg.2026.1851638)
Supplement: Supplementary file 2 [file Table2.docx]

Supplementary Table S2. Univariable Cox proportional hazards analysis for time to wound healing

| Variables | HR (95%CI) | *P* |
| --- | --- | --- |
|  |  |  |
| Group |  |  |
| Control | 1.00 (Reference) |  |
| Intervention | 2.18 (1.28 ~ 3.72) | **0.004** |
| Age | 0.95 (0.93 ~ 0.98) | **<.001** |
| Sex |  |  |
| Female | 1.00 (Reference) |  |
| Male | 0.65 (0.39 ~ 1.09) | 0.102 |
| Diabetes |  |  |
| No | 1.00 (Reference) |  |
| Yes | 0.30 (0.17 ~ 0.52) | **<.001** |
| Wound area baseline | 0.88 (0.78 ~ 0.99) | **0.047** |

Note: Univariable Cox proportional hazards regression was performed to evaluate the association between each clinical variable and time to wound healing. Hazard ratios (HRs) and 95% confidence intervals (CIs) are reported. The control group served as the reference category for treatment group comparisons. HR > 1 indicates a higher rate of wound healing.
