# Supplementary material for: Early wound closure with negative-pressure irrigation-drainage accelerates healing and improves early appearance: a comparative study
Source: Front Surg. 2026 Jul 28;13:1851638. doi: 10.3389/fsurg.2026.1851638 (PMC13457209; doi:10.3389/fsurg.2026.1851638)
Supplement: Supplementary file 3 [file Table3.docx]

Supplementary Table S3. Univariable logistic regression analysis for wound healing at 14 and 28 days

| Outcome | Variables | OR (95%CI) | *P* |
| --- | --- | --- | --- |
| Healed within 14 days | Group |  |  |
|  | Control | 1.00 (Reference) |  |
|  | Intervention | 3.13 (0.93 ~ 10.47) | 0.065 |
|  | Age | 0.91 (0.84 ~ 0.98) | **0.013** |
|  | Diabetes |  |  |
|  | No | 1.00 (Reference) |  |
|  | Yes | 0.00 (0.00 ~ Inf) | 0.992 |
|  | Wound area baseline | 0.65 (0.49 ~ 0.86) | **0.002** |
| Healed within 28 days | Group |  |  |
|  | Control | 1.00 (Reference) |  |
|  | Intervention | 9.67 (1.13 ~ 82.83) | **0.038** |
|  | Age | 0.93 (0.87 ~ 0.99) | **0.016** |
|  | Diabetes |  |  |
|  | No | 1.00 (Reference) |  |
|  | Yes | 0.24 (0.04 ~ 1.25) | 0.089 |
|  | Wound area baseline | 0.95 (0.70 ~ 1.29) | 0.742 |

Note: Univariable logistic regression analyses were conducted to assess the association between individual clinical variables and wound healing at 14 and 28 days. Odds ratios (ORs) and 95% confidence intervals (CIs) are presented. The control group served as the reference category. OR > 1 indicates a higher likelihood of wound healing at the specified time point.
